# New Advances in the Development of New Drugs and Treatment Targets for Brain Cancers (2nd Edition)

**DOI:** 10.3390/brainsci16050543

**Published:** 2026-05-21

**Authors:** Luis Exequiel Ibarra, Laura Natalia Milla Sanabria, Nuria Arias-Ramos

**Affiliations:** 1 Instituto de Biotecnología Ambiental y Salud (INBIAS), Universidad Nacional de Rio Cuarto (UNRC) y Consejo Nacional de Investigaciones Científicas y Técnicas (CONICET), Rio Cuarto X5800BIA, Argentina; lmilla@exa.unrc.edu.ar; 2 Departamento de Biología Molecular, Facultad de Ciencias Exactas, Fisicoquímicas y Naturales, Universidad Nacional de Rio Cuarto, Rio Cuarto X5800BIA, Argentina; 3 Instituto de Investigaciones Biomédicas Sols-Morreale, Consejo Superior de Investigaciones Científicas-Universidad Autónoma de Madrid (CSIC-UAM), 28029 Madrid, Spain; narias@iib.uam.es

Primary tumors of the central nervous system (CNS) represent a major clinical challenge worldwide due to their high morbidity, aggressive biological behavior, and limited therapeutic options. Among them, glioblastoma (GBM) remains the most common and lethal malignant brain tumor in adults, characterized by rapid progression, extensive infiltration into surrounding brain tissue, and a median survival that rarely exceeds 15 months despite multimodal treatment strategies including surgery, radiotherapy, and chemotherapy [1]. The complexity of brain tumors arises from multiple factors, including pronounced intratumoral heterogeneity, adaptive therapeutic resistance, and the presence of biological barriers such as the blood–brain barrier (BBB), which significantly limits the delivery of many therapeutic agents to the tumor site [2].

Over the past decade, advances in molecular biology, systems biology, and translational oncology have considerably expanded our understanding of the cellular and molecular mechanisms underlying brain tumor development and progression. These insights have stimulated the search for novel therapeutic targets and the development of innovative treatment strategies that move beyond conventional cytotoxic approaches [3,4]. Emerging research areas include targeted molecular therapies, metabolic reprogramming, immunomodulation of the tumor microenvironment, drug repurposing strategies, and advanced delivery systems capable of improving therapeutic penetration into the CNS [5,6]. In parallel, progress in imaging technologies, radiotheranostics, and computational drug discovery approaches is contributing to a more integrated and personalized framework for brain tumor treatment [7,8].

Despite these important advances, significant knowledge gaps remain regarding the mechanisms that drive tumor progression, therapy resistance, and interactions between tumor cells and the surrounding microenvironment [9,10]. Addressing these challenges requires multidisciplinary efforts that combine expertise in neuro-oncology, pharmacology, nanomedicine, molecular biology, and computational biology. In this context, the Special Issue entitled “New Advances in the Development of New Drugs and Treatment Targets for Brain Cancers (2nd Edition)” was conceived to provide an updated overview of emerging therapeutic strategies and to highlight innovative research addressing the biological and translational challenges associated with brain tumors.

This Special Issue gathers contributions that explore diverse aspects of brain tumor biology and therapy development, with a particular emphasis on GBM. The published articles encompass several complementary research directions, including the identification of new molecular targets involved in tumor progression, the development of targeted therapeutic agents, the exploration of drug repurposing strategies, and the evaluation of natural or bioactive compounds with potential antitumor activity. In addition, the issue highlights advances in precision medicine approaches aimed at improving diagnostic accuracy and therapeutic specificity through the integration of molecular targeting and imaging technologies.

A number of contributions address the identification and validation of novel therapeutic targets involved in key signaling pathways that regulate tumor growth, metabolism, and cell survival. Understanding the molecular drivers of glioma progression remains essential for the development of targeted therapies capable of overcoming the intrinsic resistance mechanisms that characterize these tumors. Several studies included in this Special Issue explore how the modulation of specific signaling pathways or metabolic processes can influence tumor cell proliferation, apoptosis, and interactions with the tumor microenvironment.

Another important theme emerging from this Special Issue is the growing interest in drug repurposing as an efficient strategy for accelerating the discovery of new therapeutic options for brain cancers. Because many approved drugs already possess well-characterized pharmacokinetic and safety profiles, repurposing strategies can significantly reduce the time and cost associated with drug development. Computational approaches combined with experimental validation are increasingly being used to identify promising candidate compounds that may exhibit antitumor activity in glioblastoma and other brain tumors.

The role of tumor metabolism also represents a key area of investigation highlighted in this collection. Metabolic reprogramming is a hallmark of cancer and plays a critical role in supporting tumor growth, survival, and resistance to therapy. Brain tumors, particularly GBM, exhibit profound alterations in glucose metabolism and energy production pathways. Targeting metabolic vulnerabilities therefore represents a promising therapeutic strategy that may complement existing treatments.

Furthermore, several contributions examine the potential of bioactive natural compounds and small molecules as modulators of tumor signaling pathways and inflammatory responses within the tumor microenvironment. Natural compounds continue to attract considerable interest in oncology research due to their diverse biological activities and potential to influence multiple cellular pathways simultaneously. Such approaches may offer new opportunities for combination therapies aimed at improving therapeutic efficacy while reducing toxicity.

Another emerging area highlighted in this Special Issue involves advanced imaging and theranostic strategies, which integrate diagnostic and therapeutic capabilities into a single platform. These approaches hold considerable promise for improving the precision of tumor detection and enabling the targeted delivery of therapeutic agents directly to tumor tissues. By combining molecular targeting with imaging technologies, theranostic platforms may contribute to more personalized treatment strategies in neuro-oncology.

Collectively, the contributions included in this Special Issue reflect the rapidly evolving landscape of brain tumor research and underscore the importance of multidisciplinary collaboration in the development of next-generation therapies. The studies presented here provide valuable insights into the molecular and cellular mechanisms underlying brain tumor biology and highlight innovative strategies that may contribute to improving treatment outcomes in the future.

Looking ahead, several key directions should be considered in future research efforts. First, there is a growing need to integrate molecular profiling, computational modeling, and functional validation approaches to better identify clinically relevant therapeutic targets. Second, the development of advanced drug delivery systems capable of effectively crossing the BBB remains a major priority in neuro-oncology. Third, improved preclinical models that more accurately recapitulate the complex tumor microenvironment of brain tumors will be essential for translating experimental discoveries into clinical applications.

In conclusion, this Special Issue provides a comprehensive overview of recent advances in the development of new therapeutic strategies and targets for brain cancers. The findings presented here highlight the progress being made in understanding the molecular complexity of these tumors and emphasize the importance of innovative and interdisciplinary approaches to address the persistent challenges in neuro-oncology. Continued efforts in this field are expected to contribute to the development of more effective and personalized therapies that ultimately improve survival and quality of life for patients with brain tumors.
